# FastGGM: An Efficient Algorithm for the Inference of Gaussian Graphical Model in Biological Networks

**DOI:** 10.1371/journal.pcbi.1004755

**Published:** 2016-02-12

**Authors:** Ting Wang, Zhao Ren, Ying Ding, Zhou Fang, Zhe Sun, Matthew L. MacDonald, Robert A. Sweet, Jieru Wang, Wei Chen

**Affiliations:** 1 Division of Pulmonary Medicine, Allergy and Immunology; Department of Pediatrics, Children’s Hospital of Pittsburgh of UPMC, University of Pittsburgh, Pittsburgh, Pennsylvania, United States of America; 2 Department of Statistics, University of Pittsburgh, Pittsburgh, Pennsylvania, United States of America; 3 Department of Biostatistics, University of Pittsburgh Graduate School of Public Health, Pittsburgh, Pennsylvania, United States of America; 4 Department of Psychiatry, University of Pittsburgh, Pittsburgh, Pennsylvania, United States of America; 5 Department of Neurology, University of Pittsburgh, Pittsburgh, Pennsylvania, United States of America; 6 VISN 4 Mental Illness Research, Education and Clinical Center (MIRECC), VA Pittsburgh Healthcare System, Pittsburgh, Pennsylvania, United States of America; 7 Department of Human Genetics, University of Pittsburgh Graduate School of Public Health, Pittsburgh, Pennsylvania, United States of America; Microsoft Research, UNITED STATES

## Abstract

Biological networks provide additional information for the analysis of human diseases, beyond the traditional analysis that focuses on single variables. Gaussian graphical model (GGM), a probability model that characterizes the conditional dependence structure of a set of random variables by a graph, has wide applications in the analysis of biological networks, such as inferring interaction or comparing differential networks. However, existing approaches are either not statistically rigorous or are inefficient for high-dimensional data that include tens of thousands of variables for making inference. In this study, we propose an efficient algorithm to implement the estimation of GGM and obtain p-value and confidence interval for each edge in the graph, based on a recent proposal by Ren et al., 2015. Through simulation studies, we demonstrate that the algorithm is faster by several orders of magnitude than the current implemented algorithm for Ren et al. without losing any accuracy. Then, we apply our algorithm to two real data sets: transcriptomic data from a study of childhood asthma and proteomic data from a study of Alzheimer’s disease. We estimate the global gene or protein interaction networks for the disease and healthy samples. The resulting networks reveal interesting interactions and the differential networks between cases and controls show functional relevance to the diseases. In conclusion, we provide a computationally fast algorithm to implement a statistically sound procedure for constructing Gaussian graphical model and making inference with high-dimensional biological data. The algorithm has been implemented in an R package named “FastGGM”.

## Author Summary

Gaussian graphical model (GGM), a probability model for characterizing conditional dependence among a set of random variables, has been widely used in studying biological networks. It is important and practical to make inference with rigorous statistical properties and high efficiency under a high-dimensional setting, which is common in biological systems that usually contain tens of thousands of molecular elements, such as genes and proteins. This work proposes a novel efficient algorithm, FastGGM, to implement asymptotically normal estimation of large GGM established by Ren et al [1]. It quickly estimates the precision matrix, partial correlations, as well as p-values and confidence intervals for the graph. Simulation studies demonstrate our algorithm outperforms the current algorithm for Ren et al. and algorithms for some other estimation methods, and real data analyses further prove its efficiency in studying biological networks. In conclusion, FastGGM is a statistically sound and computationally fast algorithm for constructing GGM with high-dimensional data. An R package for implementation can be downloaded from http://www.pitt.edu/~wec47/FastGGM.html.

## Introduction

Biological networks, involving biochemical reactions, regulatory interactions or other relationships among molecules, such as DNA, RNA and proteins, play a critical role in various biological processes. Understanding the static and dynamic structure of biological networks can help to elucidate important mechanisms of complex biological processes and diseases [2,3].

Traditional regression or co-expression models, while being widely used, can only explore marginal correlations but cannot distinguish direct or indirect (e.g. through intermediates) relationships. Graphical Model (GM) is relatively more realistic to present complex networks due to its interpretation with conditional dependence. Although GM is a classical and well-studied statistical model, with the advance of technology for data collection, biological applications impose a new challenge, in which the number of variables or features is often far larger than the sample size. Many efforts have been spent on analyzing biological networks in the past decade using GM in this high-dimensional setting under certain sparseness assumptions due to the belief that biological molecules operate in specific biological pathways and the genetic networks are intrinsically sparse. Typically, the existing methods depend on regularization techniques. Relevant methodology and theoretical works include: penalized likelihood estimation of the sparse precision matrix in Gaussian graphical model (GMM) [4]; neighborhood selection with the Lasso to estimate neighbors separately for each variable in sparse high-dimensional graphs [5]; applying the scaled Lasso to obtain optimal convergence rate in the estimation of precision matrix [6]; and others. In spite of extensive literature on the topic, statistical inference is not rigorous enough, and the precise relationship between regularized parameters and the number of false edges is unclear. Hence, Liu proposed a simultaneous testing procedure for conditional dependence in GGM to control the false discovery rate [7]. More recently, Ren et al. proposed a novel regression-based method to obtain asymptotically normal estimation of large GGM under a minimal sparseness condition [1], it provides both p-value and confidence interval for each edge in the graph. However, the computation of naïve implementation of the method in real biological applications, which often possess tens of thousands of variables, is very expensive. There are some other recent developments on inference of GGM: Janková and van de Geer applied a bias correction approach to make inference of each edge in the graph [8] based on the popular penalized likelihood estimation [4]; Gu et al. extended this idea to the high-dimensional inference of Gaussian copula graphical model [9] based on a novel decorrelated score test proposed by Ning and Liu [10]. However, both works also encounter the same implementation issue, besides some extra model assumptions, compared to Ren et al [1].

In this study, we propose an efficient algorithm named FastGGM for the inference of GGM to implement the theoretical work of Ren et al. [1] and close the gap between the theoretical development and real applications in high-dimensional settings. Using both simulated and real data sets, we demonstrate that our algorithm is able to speed up the existing implementation in several orders of magnitudes without losing efficiency in estimation. Therefore, FastGGM can make it feasible to infer biological networks under the framework of GGM at a whole-genome scale.

## Methods

We first briefly describe the Gaussian graphical model and summarize the statistical properties of the inference method proposed by Ren et al [1]. An efficient implementation algorithm is introduced for this method in the next section.

### Basic model

The methodology of this study is based on the Gaussian graphical model (GGM), a probability model that characterizes the conditional dependence structure of a set of random variables by a graph. Let *X* = (*X*_1_,*X*_2_,⋯,*X*_*p*_)′ be a multivariate Gaussian random vector with mean *μ* (we assume *μ* = 0 hereafter) and covariance matrix Σ. A GGM associated with *X* is a graph *G* = (*V*, *E*). The node set *V* = {*X*_1_,*X*_2_,⋯,*X*_*p*_} has *p* components and the edge set *E* consists of pairs (*i*, *j*), where (*i*, *j*) ∈ *E* if there is an (undirected) edge between *X*_*i*_ and *X*_*j*_. There is an edge between two nodes *X*_*i*_ and *X*_*j*_ if and only if *X*_*i*_ and *X*_*j*_ are conditional dependent given all other variables {*X*_*K*_, *k* ≠ *i*, *j*}. It is well known that the conditional independence between *X*_*i*_ and *X*_*j*_ given other variables is equivalent to that the corresponding element in the precision matrix is zero [11,12], i.e., *ω*_*ij*_ = 0, where the precision matrix is the inverse covariance matrix Ω = (*ω*_*ij*_) = Σ^−1^. Given *n* i.i.d and samples *X*^(1)^,⋯,*X*^(*n*)^, the goal is to make statistical inference of each edge in the graph, or equivalently of each *ω*_*ij*_.

In the classical low-dimensional setting, in which *p* is fixed while *n* goes to infinity, it is natural to apply the maximal likelihood estimator, the inverse of sample covariance matrix, to obtain the asymptotic normality estimation of each edge at $\sqrt{n}$ rate. However, in modern applications of biological network, the dimension *p* is often far larger than *n*, so the inverse sample covariance matrix does not exist or is inconsistent. Motivated by the sparseness assumption of the graph, i.e., most *ω*_*ij*_ are zeros, Ren et al. and Sun and Zhang tackled the inference problem from another point of view of the model [1,13].

Assume we are interested in the partial correlation between the *i*th and *j*th variables, we model the conditional Gaussian distribution with index set *A* = {*i*, *j*}, $X_{A}|X_{A^{c}}∼N\left(-\Omega_{A,A}^{-1}\Omega_{A,A^{c}}X_{A^{c}},\Omega_{A,A}^{-1}\right),\;\Omega_{A,A}=\left(\begin{array}{cc} \omega_{ii} & \omega_{ij}\\ \omega_{ij} & \omega_{jj} \end{array}\right)$, where *X*_*C*_ represents the coordinates of *X* indexed by *C* and Ω_*C*,*D*_ denotes the submatrix of Ω with rows and columns indexed by *C* and *D* respectively. This observation motivates us to consider the estimation of individual element *ω*_*ij*_ by estimating the noise level Ω_*A*,*A*_ in the bivariate regression of *X*_*A*_ against $X_{A^{c}}$. To be specific, we regress the *i*th and the *j*th columns ***X***_***A***_ of the *n* × *p* dimensional data matrix ***X*** = (*X*^(1)^,⋯,*X*^(*n*)^)′ against the remaining columns $X_{A^{c}}$ based on the equation $X_{A}=X_{A^{c}}\beta_{A}+\epsilon_{A}$, where the true coefficients $\beta_{A}\prime =-\Omega_{A,A}^{-1}\Omega_{A,A^{c}}$ is sparse due to the sparseness structure of the graph and rows of ***ϵ***_***A***_ are i.i.d. Gaussian vectors with mean zero and covariance $\Omega_{A,A}^{-1}$. A tuning-free penalized approach, scaled Lasso was used for this regression to obtain an estimator ${\hat{\beta }}_{A}$ of ***β***_***A***_ as well as the residue ${\hat{\epsilon }}_{A}=X_{A}-X_{A^{c}}{\hat{\beta }}_{A}$. Then the final estimator ${\hat{\omega }}_{ij}$ can be formally written as ${\hat{\Omega }}_{A,A}=\left(\begin{array}{cc} {\hat{\omega }}_{ii} & {\hat{\omega }}_{ij}\\ {\hat{\omega }}_{ij} & {\hat{\omega }}_{jj} \end{array}\right)={\left({\frac{1}{n}\hat{\epsilon }}_{A}^{\prime }{\hat{\epsilon }}_{A}\right)}^{-1}$. It is worthwhile to note that the method is tuning-free and thus can avoid the statistical problems arisen from the cross-validation procedure, and the estimator is proven optimal in the decision theory framework. In particular, when the network graph is sufficiently sparse, the number of active variables or non-zero edges is not too large relative to the sample size, i.e., the maximum node degree of the graph satisfies $s=o\left(\sqrt{n}/\;\text{log}\;p\right)$, besides the bounded eigenvalue condition on Ω, the estimator is asymptotically efficient in the sense that $\sqrt{nF_{ij}}\left({\hat{\omega }}_{ij}-\omega_{ij}\right)\overset{D}{\to }N\left(0,1\right),F_{ij}={\left({\hat{\omega }}_{ii}{\hat{\omega }}_{ii}+{{\hat{\omega }}_{ij}}^{2}\right)}^{-1}$. A closely related quantity, partial correlation, which has a natural interpretation for the strength of conditional dependence, is formally written as ${\hat{\gamma }}_{ij}=-{\hat{\omega }}_{ij}/\sqrt{{\hat{\omega }}_{ii}{\hat{\omega }}_{jj}}$ with the property $\sqrt{n{\left(1-{\hat{\gamma }}_{ij}^{2}\right)}^{-2}}\left({\hat{\gamma }}_{ij}-\gamma_{ij}\right)\overset{D}{\to }N\left(0,1\right)$.

Ren et al.’s theoretical study provides asymptotically normal estimation of each edge in GGM under a minimal sparseness assumption, hence can provide p-value and confidence interval for each *ω*_*ij*_ [1].

### FastGGM algorithm

Although theoretically Ren et al. have developed a justified tuning-free inference methodology [1], it is still not very clear if it can handle real high-dimensional datasets in a reasonable time. While no software was provided by Ren et al., later Chen et al. developed an R package named “ANTAC” for a naïve implementation of the methodology [14]. However, as the procedure of the method implies, in order to obtain asymptotically normal estimators of all edges, an order of *O*(*p*^2^) runs of scaled Lasso regressions need to be implemented, which make the computation with ANTAC for tens of thousands of variables extremely challenging and even infeasible.

**Fig 1:**
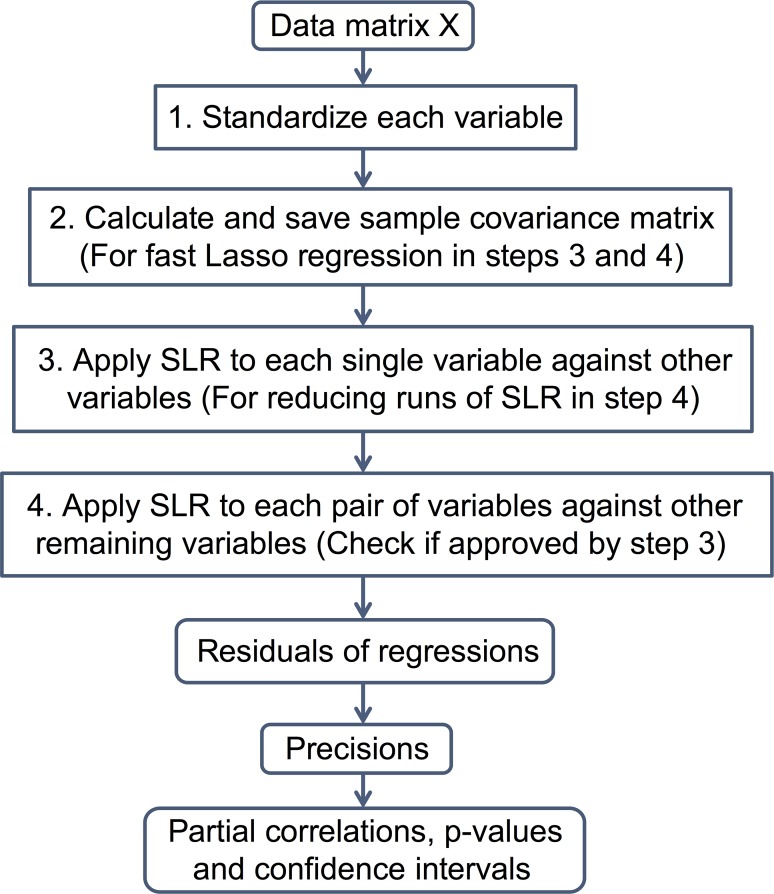
Flowchart of FastGGM algorithm.

In this study, we develop a tuning-free and efficient algorithm to accelerate the implementation and make the method of Ren et al. computationally attractive and feasible with tens of thousands of variables. Specifically, by focusing on the cyclical coordinate descent method for Lasso regression [15], our new algorithm pre-calculates and saves the sample covariance matrix which is used for each single run of scaled Lasso regression later. As a result, the new algorithm is able to avoid repetitive computation of this major step for all of *O*(*p*^2^) runs (or *O*(*sp*) runs, see Discussion below the flowchart) of Lasso and thus its computational cost is significantly reduced. Our fast algorithm is not an approximate version but provides exact solution of the method proposed by Ren et al. and thus can accurately generate partial correlation, p-value and confidence interval of the edge between any two nodes in the studied network. We outline a detailed procedure below (Fig 1), followed by some discussion on the advantage of our algorithm:

1. Standardize each variable in data matrix *X*:
  - Center each column of *X* to have mean zero and get matrix *X*_*c*;
  - Scale each column of *X*_*c* by $\sqrt{n}/\|{X\_c}_{\cdot i}\|$ for all *i* = 1,⋯,*p*, where ‖⋅‖ denotes the vector $l_{2}$ norm, and get matrix *X*_*s*;
2. Calculate sample covariance matrices for the purpose of accelerating Lasso regression using coordinate descent optimization based on covariance updates [16,17], they are:
  - *IP*_*YX*, where *IP*_*YX*_*ij*_ is the inner product between the column *i* of *X*_*c* and the column *j* of *X*_*s*;
  - *IP*_*XX*, where *IP*_*XX*_*ij*_ is the inner product between the column *i* and column *j* of *X*_*s*. It can be rapidly calculated via multiplying each column of *IP*_*YX* by $\sqrt{n}/\|{X\_c}_{\cdot i}\|$;
3. Apply scaled Lasso regression (SLR) to each variable *i* against all other variables *i*^*c*^:
  $${\text{min}}_{\beta \in R^{p-1},\sigma \in R^{+}}\left\{\frac{{\left\|{X\_c}_{i}-{X\_s}_{i^{c}}\beta \right\|}^{2}}{2n\sigma }+\frac{\sigma }{2}+\lambda \;\sum_{k\in i^{c}}|\beta_{k}|\right\}$$ (1)
  - Fix *σ* and solve a fast Lasso regression based on the pre-calculated covariance matrices to get *β* (See the pseudo code of fast Lasso solver in S1 File);
  - Fix *β* and update $\sigma =\sqrt{{\|{X\_c}_{i}-{X\_s}_{i^{c}}\beta \|}^{2}/n}$;
  - Iterate steps a and b until *σ* and *β* converge to get $\hat{\sigma }$ and $\hat{\beta }$;
  - Save $\hat{\beta }$ and residual $\hat{\epsilon }={X\_c}_{i}-{X\_s}_{i^{c}}\hat{\beta }$ for variable *i* respectively into the *i* column of a *p* × *p* matrix ***B*** = (*b*_*ij*_) with 0 diagonal and a *n* × *p* matrix $\hat{\epsilon }$;
4. Estimate conditional dependence relationship for each pair of variables (e.g. *i* and *j*, *i* ≠ *j*). Results from step 3 allow reducing computations of SLR due to the sparse assumption:
  - For the variable *i*, get residual of SLR against all other variables except variables *i* and *j* (i.e. {*i*, *j*}^*c*^):
    - If *b*_*ji*_ = 0 in the saved ***B*** matrix, directly get ${\hat{\epsilon }}_{i}$ from column *i* of the pre-saved $\hat{\epsilon }$ matrix;
    - If *b*_*ji*_ ≠ 0, apply SLR (2) in the same way as that in step 3 to obtain the residue ${\hat{\epsilon }}_{i}$;
      $${\text{min}}_{\beta \in R^{p-2},\sigma \in R^{+}}\left\{\frac{{\left\|{X\_c}_{i}-{X\_s}_{A^{c}}\beta \right\|}^{2}}{2n\sigma }+\frac{\sigma }{2}+\lambda \;\sum_{k\in A^{c}}|\beta_{k}|\right\},\;A=\{i,j\}$$ (2)
  - For variable *j*, similarly get residual of regression against variables {*i*, *j*}^*c*^:
    - If *b*_*ij*_ = 0 in the saved *B* matrix, directly get ${\hat{\epsilon }}_{j}$ from column *j* of the pre-saved $\hat{\epsilon }$ matrix;
    - If *b*_*ij*_ ≠ 0, apply SLR (3) to obtain residue ${\hat{\epsilon }}_{j}$;
      $${\text{min}}_{\beta \in R^{p-2},\sigma \in R^{+}}\left\{\frac{{\left\|{X\_c}_{j}-{X\_s}_{A^{c}}\beta \right\|}^{2}}{2n\sigma }+\frac{\sigma }{2}+\lambda \;\sum_{k\in A^{c}}|\beta_{k}|\right\},\;A=\{i,j\}$$ (3)
  - Calculate precision matrix for variables *i* and *j*:
    $$\left(\begin{array}{cc} {\hat{\omega }}_{ii} & {\hat{\omega }}_{ij}\\ {\hat{\omega }}_{ij} & {\hat{\omega }}_{jj} \end{array}\right)={\left[\frac{1}{n}\left(\begin{array}{c} {\hat{\varepsilon }}_{i}^{\prime }\\ {\hat{\varepsilon }}_{j}^{\prime } \end{array}\right)(\begin{array}{cc} {\hat{\varepsilon }}_{i} & {\hat{\varepsilon }}_{j} \end{array})\right]}^{-1}$$ (4)
  - Estimate p-value and confidence interval for *ω*_*ij*_ from distribution:
    $$\sqrt{n{\left({\hat{\omega }}_{ii}{\hat{\omega }}_{jj}+{\hat{\omega }}_{ij}^{2}\right)}^{-1}}\left({\hat{\omega }}_{ij}-\omega_{ij}\right)\overset{D}{\to }N(0,\;1)$$ (5)
  - Calculate partial correlation ${\hat{\gamma }}_{ij}=-{\hat{\omega }}_{ij}/\sqrt{{\hat{\omega }}_{ii}{\hat{\omega }}_{jj}}$;
  - Estimate p-value and confidence interval for *γ*_*ij*_ from distribution:
    $$\sqrt{n{\left(1-{\hat{\gamma }}_{ij}^{2}\right)}^{-2}}\left({\hat{\gamma }}_{ij}-\gamma_{ij}\right)\overset{D}{\to }N(0,\;1)$$ (6)

The advantage of our fast algorithm over the algorithm in the software developed by Chen et al. is mostly due to steps 2 and 3. First of all, step 2 is completely new and can save computation in all runs of SLR in steps 3 and 4. To see the reason, we take a close look at the covariance updates in the coordinate descent optimization for Lasso regression, which is reflected in the line 15 of the pseudo code (See S1 File). For each single run of Lasso regression, inner products of response and design matrix as well as sample covariance of the design matrix need to be computed in advance (*O*(*np*^2^) operations). Although initial computation procedures for different Lasso regressions in steps 3 and 4 are slightly different, they all rely on a single sample covariance matrix of the data matrix *X*. Realizing this fact, the number of operations can be significantly reduced from *O*(*np*^4^) (or *O*(*nsp*^3^)) to only *O*(*np*^2^) since there are *O*(*p*^2^) (or *O*(*sp*)) runs of Lasso in steps 3 and 4. Secondly, as briefly commented by Ren et al. [1], the extra *p* runs of scaled Lasso in step 3 can further significantly save the computation in step 4 and reduce the required number of runs of scaled Lasso from *O*(*p*^2^) to *O*(*sp*). This is because whenever *b*_*ji*_ = 0, the solution of scaled Lasso applied to the *i*th variable against all others (saved in step 3) is equal to that of SLR in (2). Since the maximum node degree is *s*, we expect there are no more than *s* nonzero elements in each column of ***B***.

Besides the fast algorithm, we develop FastGGM package with R language and two libraries, “Rcpp” [18] and “RcppParallel”, which help implement C++ functions in R and significantly speed up the loop operation. The package has three main functions: “FastGGM” is for analyzing GGM with one CPU, “FastGGM_parallel” is for analyzing with parallel computation, and “FastGGM_pairs” is for analyzing the conditional dependence for specific variable pairs. The first two functions output matrices of precision and partial correlation as well as the corresponding p-value and confidence interval for every pair of variables. The last function outputs vectors of precision, partial correlation, p-value, and confidence interval for the specified variable pairs. According to Section 5.1 in Ren et al.’s work, the default value of penalty parameter *λ* in our package is set as $\sqrt{\left(2\times log\left(p/\sqrt{n}\right)\right)/n}$.

## Results

### Simulation analysis

To illustrate the efficiency of FastGGM, we simulated data as follows: Firstly, we generated the upper triangular of a *p* × *p* sparse precision matrix Ω with diagonal element being 4, where the probability of each off-diagonal element being nonzero was *π* and its value was randomly set as 0.3, 0.6 or 1 with equal probability whenever the element is nonzero. Next, we made Ω symmetrical and set the elements of its bottom right block, whose size was $\frac{p}{2}\times \frac{p}{2}$, as twice of the elements of its top left $\frac{p}{2}\times \frac{p}{2}$ block. Finally, we inversed Ω to get a covariance matrix, and generated a *n* × *p* data matrix *X* by randomly sampling from a multivariate Gaussian distribution *N*(0, Ω^−1^). Note that Ω is invertible with high probability by our construction. Eleven models with different combinations of parameters {*π*, *p*, *n*} were considered in our data simulation (See Table 1).

**Table 1:** Performance of estimating the precision matrix.

*Π*	*p*	*n*	PCC	ChebDist	Type I error (p-value < 0.01)	AUC
0.04	100	400	0.946	1.793	0.0088	0.878
0.02	200	400	0.903	1.938	0.0092	0.872
0.01	400	100	0.592	4.925	0.0073	0.721
0.01	400	200	0.725	3.023	0.0086	0.806
0.01	400	400	0.832	1.995	0.0093	0.879
0.01	400	800	0.905	1.356	0.0095	0.936
0.005	800	400	0.730	2.107	0.0093	0.886
0.005	1000	800	0.806	1.423	0.0096	0.941
0.0025	2000	800	0.695	1.520	0.0096	0.946
0.0001	5000	800	0.523	1.608	0.0097	0.946
0.0005	10000	1000	0.436	1.462	0.0097	0.960

Next, we estimated the underlying precision matrices from the simulated data matrices. For each precision matrix, we randomly generated 100 data matrices and respectively applied FastGGM on them. Then we compared differences between the true precision matrix and 100 estimated ones. We calculated Pearson correlation coefficient (PCC) and Chebyshev distance (ChebDist, defined as the greatest difference along any coordinate dimension) between two vectors consisting of the upper triangular entries (because the matrices are symmetric) to measure the differences. Ideally, a good estimation should present large PCC and small ChebDist. Table 1 shows the mean differences of 100 estimations for each precision matrix. It is shown that when *p* is fixed (e.g. 400) and *n* increases, PCC gets larger and ChebDist gets smaller; while *n* is fixed (e.g. 400 or 800) and *p* increases, PCC turns smaller and ChebDist becomes larger. This means that data with more samples or less variables are better for the inference of GGM. Besides, we evaluated the estimation of nonzero entries in the precision matrix, which could be considered as conditional interactions between the variables. We calculated mean type I error of 100 estimations at the level of 0.01 using the output p-values. The results in Table 1 show that FastGGM controls type I error well. In addition, we generated Receiver Operating Characteristic (ROC) curve, which presents the true-positive rate against false-positive rate at various threshold settings, using the estimated p-values and the truth of zero and non-zero entries in the simulated precision matrix, and then computed the Area Under the Curve (AUC) to measure the performance of estimation. The larger AUC represents the better estimation. Table 1 also lists the mean AUC of 100 estimations for each precision matrix model, all AUCs are large enough to demonstrate that the estimations are accurate, and when *p* is fixed AUC consistently goes higher as *n* increases, which again indicates that large sample size is beneficial to the estimation of conditional interaction relationship among variables. Note that Ren et al. had compared the estimator of the entire sparse precision matrix via the current available algorithm after certain data-driven thresholding procedure with the existing $l_{1}$ penalized methods, such as GLasso and CLIME, and reported better performance [1], we additionally compared with another Spectral method proposed by Honorio and Jaakkola with an $l_{2}$ penalty [19]. As a result, FastGGM outperformed Honorio’s method on estimating the sparse precision matrix after the thresholding procedure (See results in S2 File).

FastGGM is designed to accelerate the inference of GGM on large-scale data sets based on the theoretical work proposed by Ren et al [1]. Note that Chen et al. developed the R package ANTAC, in which a function ANT_GGM is a naïve implementation of the original method [14]. In simulation studies, we compared the computational time of FastGGM on each simulation data set with the ANT_GGM and another fast stock method GLasso [4] that was implemented in R package “huge” [20]. Table 2 shows that FastGGM is much faster than ANT_GGM on all data sets. In particular, our algorithm is still feasible for even *p* = 5000 or 10,000 while ANT_GGM clearly cannot handle the settings. It is worthwhile to point out that besides the structural advantages of our algorithm over the naïve one implemented by ANT_GGM, there is another important aspect for saving the computational time complexity: we implemented C++ functions in R environment for efficient loop computation with the help of Rcpp and RcppParallel libraries. On the other hand, Table 2 also shows that the computational time of FastGGM is comparable with huge_glasso and even faster when applying parallel computing with 10 CPUs. The parallel computation is another big advantage of FastGGM and makes it more feasible in real applications. Besides, note that GLasso is only able to provide point estimation of the precision matrix without inference results, while FastGGM can additionally provide p-values and confidence intervals.

**Table 2:** Comparison of computational time.

*Π*	*p*	*n*	FastGGM_parallel with 10 CPUs (s)	FastGGM (s)	Huge_glasso (s)	ANT_GGM (s)
0.04	100	400	0.064	0.395	0.593	819.441
0.02	200	400	0.236	1.488	1.108	3502.05
0.01	400	100	0.281	1.465	6.213	13780.36
0.01	400	200	0.516	2.735	6.406	15818.482
0.01	400	400	1.079	6.095	4.829	20306.049
0.01	400	800	3.14	20.876	4.139	31023.622
0.005	800	400	7.565	50.136	30.542	202324.949
0.005	1000	800	33.083	123.483	50.366	576136.41
0.0025	2000	800	175.816	922.169	457.981	3196656.58
0.0001	5000	800	5799.889	9902.518	8431.532	-
0.0005	10000	1000	48702.67	74007.22	59084.709	-

### Global gene association networks in childhood asthma

We applied our algorithm on a public gene expression microarray data set, E-MTAB-1425 [21], which was measured from the human lymphoblastoid cells of 258 asthmatic children and 134 healthy children, to estimate the global gene association networks in normal and disease cells and compare the differences between the two networks.

**Fig 2:**
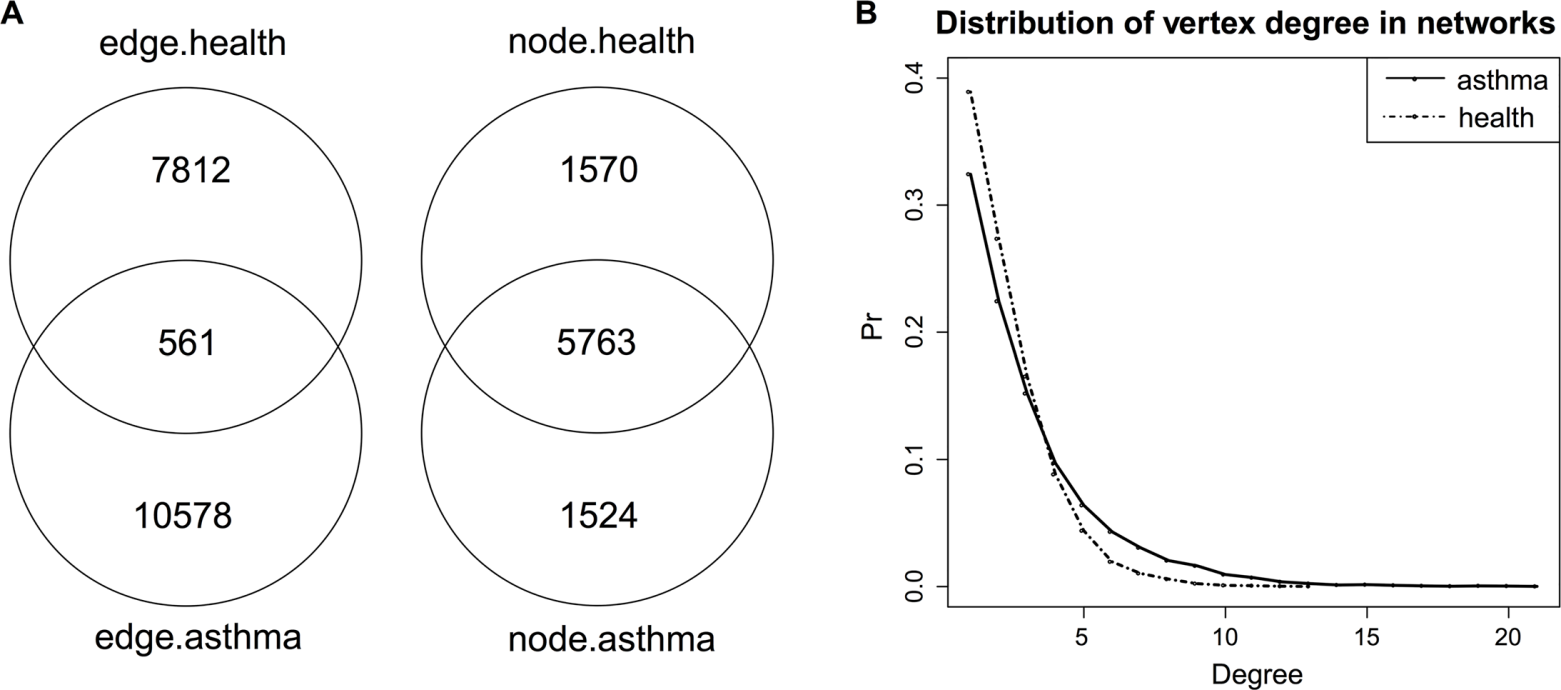
Comparing gene association networks under asthmatic and healthy conditions. A) Venn diagram of the edges and nodes in the asthmatic and healthy networks. B) Distributions of vertex degree in the two networks.

We downloaded the normalized data from the EMBL-EBI ArrayExpress database [22], and averaged the expression values of probes that represented the same genes in each sample. Then we removed genes that had a mean or standard deviation ranked below 40%, and remained the expression data of 10,049 genes. Next, we filtered 111 genes that were highly associated with either age or gender (FDR < = 0.01 under univariate regression), and generated two expression matrices of 9,938 genes for the disease and normal samples. After this preprocessing, we respectively utilized FastGGM on the two matrices to estimate the corresponding GGMs of genes. From the results we selected significant gene pairs, whose partial correlations had FDR < = 0.01, and constructed the gene association networks under the two conditions. A Venn diagram of the edges and nodes in the two networks (Fig 2A) showed that the disease network had more edges than the healthy network (11,139 vs 8,373), and only 561 edges were overlapped, although there was a large proportion of nodes overlapped between the two networks. These indicated the associations among genes changed a lot when the disease occurred. Comparing the vertex degrees (i.e. the number of nearest neighbors of a vertex) of the two networks, we found that the disease network had more genes with large degrees (Fig 2B). Besides, the disease network consisted of 276 clusters, which represented the maximal connected sub-networks, while the healthy network had 453 clusters. These observations indicated that the connection of genes in disease samples was stronger than that in healthy samples.

**Fig 3:**
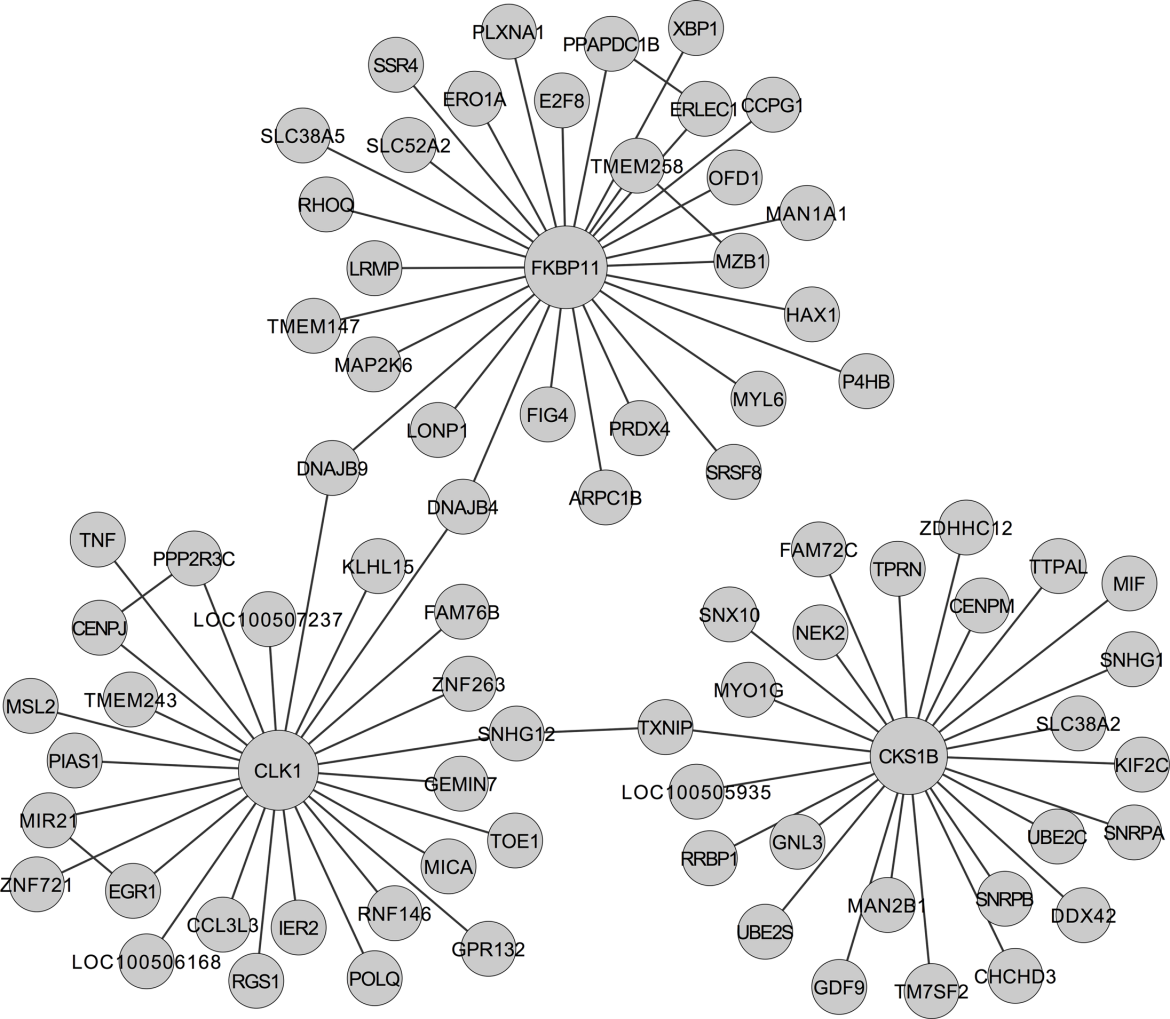
Sub-network of differential gene-gene associations between asthmatic and healthy conditions. The sizes of nodes are proportional to their degrees.

Analyzing differences of gene networks between the asthmatic and healthy conditions could be helpful for understanding the genetic mechanisms of the disease. Therefore, we merged 18,390 unique edges that only belonged to one network to construct a differential gene association network. For simplification and visualization, we selected the top 3 hub genes and extended with their nearest neighbor genes to generate a differential sub-network, which included 80 genes and 84 edges (Fig 3). In this network, many genes have been reported relevant to asthma. For example, *TNF*/*TNF-α* is a pro-inflammatory cytokine that has been implicated in many aspects of the airway pathology in asthma, and anti-*TNF-α* has been demonstrated as a potential therapy in severe refractory asthma [23]; *MIF* plays important function in the immune pathogenesis of asthma via the promotion of *TH2* responses, and its inhibition may be therapeutically beneficial to asthma [24]; The hub gene *CLK1* has been shown significantly down regulated in alveolar macrophages by diesel exhaust particles, which can induce or aggravate pulmonary diseases including asthma [25]. In addition, functional analysis on this sub-network indicated the most significantly enriched KEGG pathway of these inclusive genes was “Protein processing in endoplasmic reticulum” (Fisher’s exact test, p-value = 2e-8). It has been approved that endoplasmic reticulum stress and the related signaling networks are important modulators of inflammatory and immune responses in the development of allergen-induced bronchial asthma, especially severe asthma [26]. In short, our analysis on the gene expression data of asthma manifests that FastGGM is useful for studying large-scale biological networks.

### Synaptic protein networks in Alzheimer’s disease (AD)

Then we applied our algorithm on a proteomic data set coming from 59 AD subjects with mild to moderate dementia severity. The objective of this study was to use targeted mass spectrometry to determine levels of a number of synaptic proteins and to construct networks of these proteins. Post-mortem tissue samples from the dorsolateral prefrontal cortex (DLPFC) of the subjects were obtained from the University of Pittsburgh Alzheimer Disease Research Center Brain Tissue Bank. It was of interest to examine the synaptic protein networks for the AD subjects and to detect modules of highly interconnected proteins from the network to elucidate the disease physiology.

Protein from gray matter homogenates were assayed in triplicate using targeted mass spectrometry with a stable isotope labeled mammalian brain standard to quantify 283 peptides from 192 synaptically expressed proteins. In the data processing step, for each protein, a protein-level measure was derived by calculating the weighted average of all standardized peptide measures mapped to that protein, where the weights are inversed to the percent of coefficient of variance (CV) of the peptide measures. S1 Table provides the protein-level data for these 59 AD subjects (protein names were coded). We then applied FastGGM to construct a partial-correlation-based protein network for all the 59 AD subjects. For each pair of proteins, we tested whether the partial correlation was zero or not. As a comparison, we also performed marginal-correlation-based analysis to examine the co-expression network of the genes or proteins using the weighted gene co-expression network approach (WGCNA) [27].

We used bootstrap aggregation suggested by Schafer et al. to achieve more reliable and stable estimation [28], since the sample size in this case is small (n = 59). Specifically, we bootstrapped 200 times (with replacement) and computed the graph using FastGGM for each bootstrap. We then averaged over the 200 graphs and obtained a “bagged” partial correlation matrix for the AD group. We then tested whether the partial correlation is zero or not for each pair of proteins. In addition, we performed module detection using the hierarchical clustering approach based on partial-correlation-based network matrix and marginal-correlation-based network matrix, respectively.

**Fig 4:**
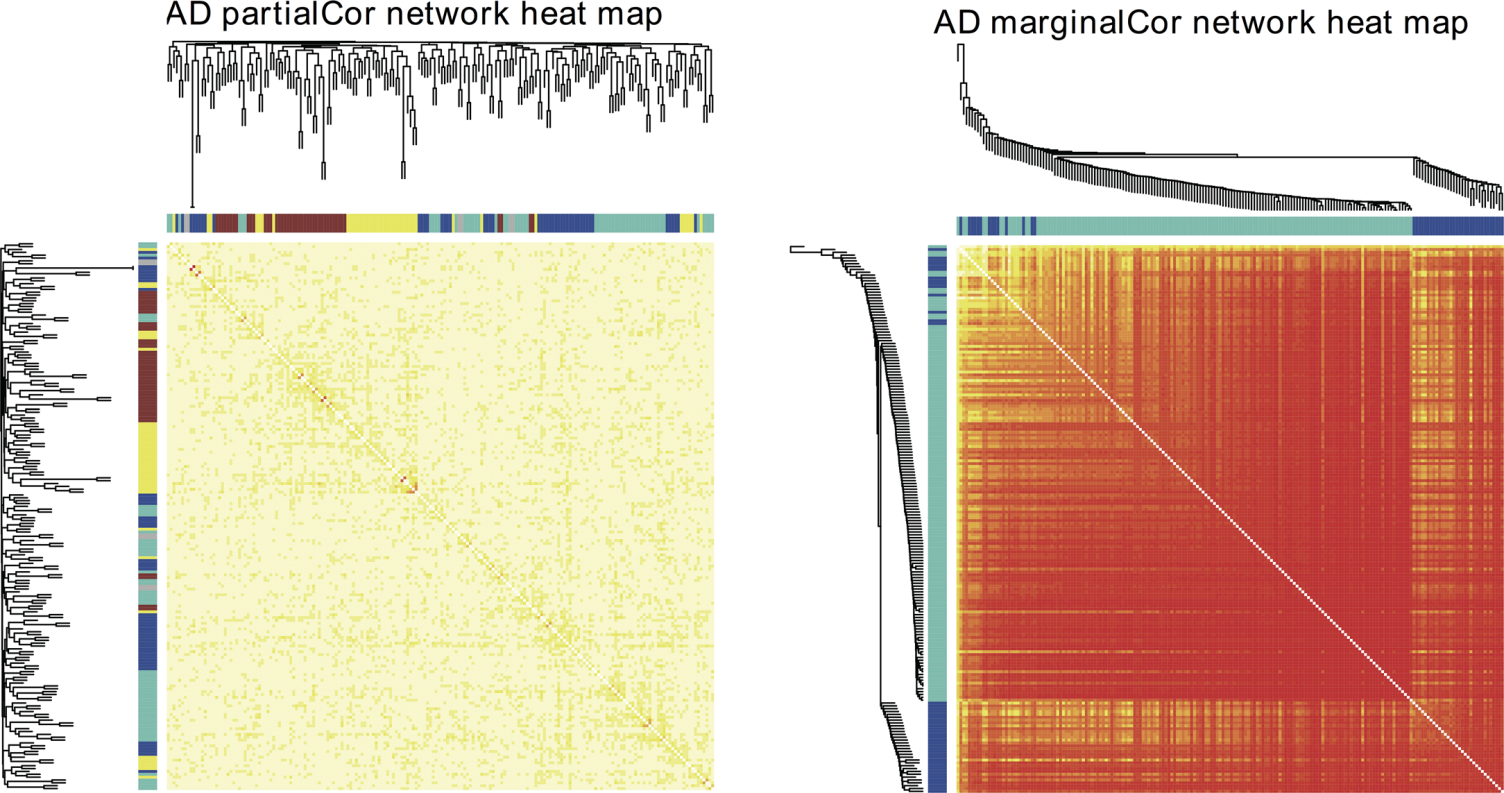
Heat maps of synaptic protein network in AD cohort where red indicates stronger correlation and the white indicates weaker correlation. The left and top color bars indicate the module membership of each protein (grey colored proteins do not belong to any module), with the corresponding hierarchical clustering dendrograms plotted. The left is the heat map based on partial correlations and the right is the heat map based on marginal correlations.

Fig 4 presents the heat map of the partial-correlation-based network constructed through FastGGM and the heat map of the marginal-correlation-based network constructed through WGCNA. We selected the same parameters, such as the soft power and minimum module size, in both network construction and module detection. With the marginal correlation approach, most of these synaptic proteins are found strongly connected (reflected by the overall red block in the heat map). There are two modules being detected. However since the entire correlations are so strong, these two modules are not really separable. In contrast, with the partial correlation approach, the overall network is much sparser. There are four modules being detected. Since the partial-correlation-based network quantifies the correlation between each pair of proteins with the effects of other proteins excluded, it only keeps the correlations due to direct causal relationships between the protein pairs, while the correlations originated via intermediate proteins are eliminated. This provides more useful information in elucidating the disease physiology, especially in the case when there are strong marginal correlations among most of the proteins so that the true underlying causal correlations are undistinguished.

With a FDR threshold of 0.001, 62 pairs of proteins are identified to have significant partial correlations, while 15289 pairs have significant marginal correlations. Table 3 lists the top 10 pairs of proteins that have the most significant FDRs of partial correlations, and the marginal correlations as well as corresponding p-values and FDR values are also listed for comparisons. It is interesting to point out that although most protein pairs with significantly strong partial correlations also have strong marginal correlations, there is a special pair, *SLC25A3* ~ *VGLUT3*, which have strong negative partial correlation but weak marginal correlation. Actually, *VGLUT3*, which is also known as *SLC17A8*, belongs to solute carrier transporter superfamily and transports substrates in cells like *SLC25A3*. And the genomic locations of the two protein genes are very close on chromosome 12. These proteins are worthy of additional verifications for their interaction and functions in the AD physiology. A simple statistical explanation of this interesting phenomenon, that two variables have significant partial correlation but non-significant marginal correlation, could be like this: some confounders affect the correlation between the two variables through other variables, after adjusting for the confounders, i.e. conditioning on other variables, their correlation may be amplified.

**Table 3:** Top 10 pairs of proteins with significant partial correlations from the AD study.

Protein1	Protein2	parCor	p.parCor	fdr.parCor	marCor	p.marCor	fdr.marCor
*CAMK2A*	*PSMA1*	0.93	0	0	0.99	1.50E-53	6.80E-50
*RAB10*	*RAB1A*	0.83	3.50E-88	3.20E-84	0.98	2.80E-42	1.40E-39
*MDH1*	*MDH2*	0.81	1.60E-76	9.70E-73	0.99	1.20E-49	3.60E-46
*RAB3A*	*SLC25A3*	0.78	2.20E-53	1.00E-49	0.92	1.40E-24	2.90E-23
*SLC25A3*	*VGLUT3*	-0.76	2.30E-44	8.40E-41	0.0039	0.98	0.98
*SLC25A3*	*SLC25A5*	0.74	1.00E-35	3.10E-32	0.93	3.40E-26	9.50E-25
*FLOT1*	*FLOT2*	0.73	7.70E-32	2.00E-28	0.88	8.80E-20	7.50E-19
*VDAC1*	*VDAC2*	0.71	1.10E-28	2.50E-25	1	4.80E-59	8.80E-55
*AP2A2*	*AP2B1*	0.66	9.00E-19	1.80E-15	0.91	3.20E-23	5.20E-22
*AP2A1*	*AP2B1*	0.64	2.10E-16	3.90E-13	0.97	1.60E-37	3.30E-35

The new algorithm finished GGM estimation over all 200 bootstraps with 4 CPUs in about 10 minutes. This example demonstrates that in the situation where the sample size and/or the number of variables are small, our FastGGM is still attractive, especially when resampling techniques are used with a large number of Gaussian graphic model estimations being performed.

## Discussion

Our fast algorithm provides an opportunity for researchers to study large-scale networks, such as gene/protein networks, using GGM in practice. Indeed, it is an exact implementation to the asymptotically normal and efficient estimation established by Ren et al [1], and hence is statistically sound. In addition, computationally the results show that the inference of partial correlation between genes becomes feasible for whole-genome data sets or small data sets that need resampling or permutations. It significantly differs from existing fast algorithms or methods on GGM that only can provide statistical estimation of the edge strength between graph nodes without the ability to do statistical inference.

There are several limitations of our method. First, the theoretical property of the method relies on the sparseness assumption of precision matrix, that is, the maximum node degree $s=o\left(\sqrt{n}/\text{log}\;p\right)$. While in many biological applications this assumption makes a lot of sense, it is impossible to obtain confidence interval with length $O\left(1/\sqrt{n}\right)$ if the sparseness assumption is violated no matter what method is applied (See details in [1]). Secondly, our inference result of conditional dependence relies on the Gaussian assumption on the data, although the inference of partial correlation may be still valid for slightly general distributional assumptions.

Motivated by other biological questions, we can extend our method on several aspects. First, It is generally believed that the architecture of cellular interactome can be re-wired under different conditions, such as environment, tissue or disease. Analyzing differential networks between conditions can help to elucidate the molecular mechanisms of complex biological processes [3]. This method will be especially helpful for identifying novel biomarkers or biological pathways for further experimental evaluation to uncover the regulation of multifactor and chronic diseases such as cancer. In our analysis on the asthma data, we respectively built two global gene networks for the disease and healthy samples under the GGM framework with a FDR threshold for determining the existence of edges, and then compared the topological changes with the unique edges that belonged to only one of the networks. Although some functional interactions with relevance to the disease were identified, this differential analysis could be improved by some rigorous statistical tests for the difference of conditional dependence over different conditions. Moreover, we believe that larger sample sizes of the cases and controls will increase the power of differential tests and promote the applications. Secondly, as pointed out, noisy genes in the set of conditional variables may introduce spurious dependencies and consequently false edges in the estimated gene networks [29,30], hence selecting a proper set of variables on which the correlation is conditioned is critical in the study of high-dimensional GGM. Thirdly, the rapid advances in data generation technologies have been producing large amounts of omic data of biological systems, such as the genetic, transcriptomic, proteomic, epigenomic, and phenomic data. The integrative analysis of the multi-omic data helps generate systematic insights into mechanisms of complex biological processes and diseases, filter the false positives introduced by the heterogeneous data sources, and provide meaningful candidate markers for further studies. Among various analytical approaches, graphical model has become a popular model to handle the heterogeneous data. For example, a few papers utilized GGM on the genetic effects gene expression quantitative loci (eQTL) data to study the conditional dependence among a set of gene expressions adjusting for genetic effects and demonstrated that the model led to more interpretable gene networks than standard GGM based on gene expression data alone [14,31–33]; some literature adopted GGM to jointly analyze the microRNA-mRNA, copy number-mRNA-methylation dependencies and their associations with clinical outcomes [34–36]. These areas are of great meaning and interest, yet beyond the scope of the current study, we will explore in future studies. Furthermore, we will collaborate with biologists to experimentally validate important findings by manipulating target gene or protein expression and examining the expression of genes or proteins from the same biological networks based on known interaction between target gene and co-expressed genes.

In conclusion, we developed a novel efficient algorithm, FastGGM, for the statistical inference of Gaussian graphical model. Through the simulation studies, we demonstrated that our new algorithm is able to speed up the current available algorithm in several orders of magnitudes without losing any accuracy. Then we applied it on two real data sets and successfully constructed the global gene/protein networks for the diseases. FastGGM can become a powerful tool of network analysis under the framework of conditional dependence, especially for high-dimensional biological data. It has been implemented in an R package, which can be downloaded from http://www.pitt.edu/~wec47/FastGGM.html.

## Supporting Information

S1 File Pseudo code of fast Lasso regression using coordinate descent based on covariance updates. (PDF)

S2 File Comparing FastGGM with Honorio’s Spectral method on estimation of the sparse precision matrices in simulations. (PDF)

S1 Table Coded protein level data in AD study. (XLSX)
