# Supplementary material for: FastGGM: An Efficient Algorithm for the Inference of Gaussian Graphical Model in Biological Networks
Source: PLoS Comput Biol. 2016 Feb 12;12(2):e1004755. doi: 10.1371/journal.pcbi.1004755 (PMC4752261; doi:10.1371/journal.pcbi.1004755)
Supplement: S1 File — (PDF) [file pcbi.1004755.s001.pdf]

S1 File. Pseudo code of fast Lasso regression using coordinate descent based on covariance updates

Problem: $\arg\min_\beta \left\{\frac{\|y-X\beta\|^2}{2N} + \lambda \sum_k |\beta_k|\right\}$

Input:

ipy: Inner product vector, $ipy_i =< y, X_{\cdot i} >$

ipx: Inner product matrix, $ipx_{ij} =< X_{\cdot i}, X_{\cdot j} >$

λ: Penalty parameter

N: Number of samples

Output:

$beta$: Regression parameter vector

```
function FastLasso(ipy, ipx, λ, N)
    stop_thr                                   # Threshold for stopping iteration
    p ← length(ipy)
    beta ← 0 with length p
    gc ← 0 with length p                       # Gradient component vector
    do
        difBeta_max ← 0
        for j = 1 → p do
            z ← (ipy[j] − gc[j])/N + beta[j]
            beta_tmp ← max(0, z − λ) − max (0, −z − λ)
            difBeta ← beta_tmp − beta[j]
            difabs ← abs(difBeta)
            if difabs > 0 then
                beta[j] ← beta_tmp
                gc ← gc + ipx[, j] × difBeta   # Update gradient components
                difBeta_max = max(difBeta_max, difabs)
            end if
        end for
    while difBeta_max ≥ stop_thr
    end do-while
    return beta
end function
```
