# Supplementary material for: FastGGM: An Efficient Algorithm for the Inference of Gaussian Graphical Model in Biological Networks
Source: PLoS Comput Biol. 2016 Feb 12;12(2):e1004755. doi: 10.1371/journal.pcbi.1004755 (PMC4752261; doi:10.1371/journal.pcbi.1004755)
Supplement: S2 File — (PDF) [file pcbi.1004755.s002.pdf]

## Comparing FastGGM with Honorio's Spectral method on estimation of the sparse precision matrices in simulations

Honorio J. and Jaakkola T. (2013) proposed a Spectral method for inverse covariance estimation for high-dimensional data in linear time and space with an $\ell_2$ penalty. FastGGM does not give the final estimation of the whole precision matrix as Honorio's method does. Indeed, an extra fully data-driven thresholding procedure is needed to obtain the final estimation of the sparse precision matrix. This final estimator is called ANT in Ren et al (2015). To have a more fair comparison, we compare ANT with Honorio's method. Specifically ANT is constructed as follows. For each entry $\widehat{\omega}_{ij}$ of precision matrix obtained from FastGGM, we recover it (keep its value) if $|\widehat{\omega}_{ij}| \geq 2\sqrt{2\xi(\widehat{\omega}_{ii}\widehat{\omega}_{jj} + \widehat{\omega}_{ij}^2)\log p/n}$ according to Ren et al (2015) with $\xi = 1$, otherwise we set it as zero. We also apply Honorio's codes (downloaded from https://www.cs.purdue.edu/homes/jhonorio/) on our simulated data sets. To compare the performances of estimating the sparse precision matrices, we separately calculate Pearson correlation coefficient (PCC, the larger the better) and Chebyshev distance (Chebdist, the smaller the better) between the estimated values and the true values of non-zero entries in the simulated matrices, and calculate mean and standard deviation (SD) of the estimated values of zero entries in the simulated matrices. Results in the following table indicate that ANT is far better on both non-zero and zero entries. In practical application of FastGGM, users can also use the output p-values for the thresholding procedure.

| $\pi$ | $p$ | $n$ | Methods | Non-zero entries | | Zero entries | |
|---|---|---|---|---|---|---|---|
| | | | | **PCC** | **Chebdist** | **Mean** | **SD** |
| 0.04 | 100 | 400 | FastGGM | 0.988 | 2 | 0 | 0 |
| | | | Honorio | 0.943 | 5.440 | -0.002 | 0.035 |
| 0.02 | 200 | 400 | FastGGM | 0.987 | 2 | 0 | 0 |
| | | | Honorio | 0.939 | 5.558 | -0.0005 | 0.030 |
| 0.01 | 400 | 100 | FastGGM | 0.970 | 6.121 | 0 | 0 |
| | | | Honorio | 0.915 | 6.304 | -0.0002 | 0.020 |
| 0.01 | 400 | 200 | FastGGM | 0.980 | 3.959 | 0 | 0 |
| | | | Honorio | 0.924 | 6.048 | -0.0001 | 0.023 |
| 0.01 | 400 | 400 | FastGGM | 0.987 | 2 | 0 | 0 |
| | | | Honorio | 0.934 | 5.665 | -7.56e-05 | 0.025 |
| 0.01 | 400 | 800 | FastGGM | 0.991 | 1.2 | 0 | 0 |
| | | | Honorio | 0.943 | 5.310 | -0.0002 | 0.026 |
| 0.005 | 800 | 400 | FastGGM | 0.985 | 2 | 0 | 0 |
| | | | Honorio | 0.928 | 5.723 | -0.0001 | 0.021 |
| 0.005 | 1000 | 800 | FastGGM | 0.989 | 2 | 0 | 0 |
| | | | Honorio | 0.939 | 5.419 | -0.0001 | 0.021 |
| 0.0025 | 2000 | 800 | FastGGM | 0.988 | 2 | 0 | 0 |
| | | | Honorio | 0.931 | 5.475 | -5.79e-05 | 0.017 |
